# Practical Medicine

**Published:** 1877-05

**Authors:** 


### II. Practical Medicine.

*Clinical Cases of Hydroadipsia. With some facts concerning the Water Supply of living Bodies.* McElroy. (*The Cincinnati Lancet and Observer*, January, 1877.)

In the Zanesville Glass Factory, the blowers are obliged to stand between two furnaces,—a melting and a heating furnace. In the warmest weather they drink from 50 to 60 pints of ice water, and some of them in addition, 2 to 3 pints of beer, in nine hours. The amount excreted by the kidneys is but little increased, nor the color of urine perceptibly changed by this enormous consumption of water. The excretions from the bowels do not seem to be increased by the use of such large quantities of drink. When not working, about 3 or 4 pints of water in 24 hours will suffice them. The amount of food consumed by them seems not augmented by labor. Most of the water drunk is eliminated by the skin; very little by the lungs, and no considerable portion by the kidneys. The men are very light in weight, hardly averaging 150 lbs. In three days they drink water equal in weight to that of their bodies. When the men lose the sense of thirst—Hydroadipsia—they cannot continue their work; but to recall the sense of thirst, salt is thrown into their mouths, which they chew and swallow, and soon the sense of thirst is restored. Dr. M. has reported quite a number of cases of Hydroadipsia; one occurring in a lady 50 years of age, who was treated by two physicians for softening of the brain. On examination of patient, the author says: Found her with continuous pain in the side, sick at the stomach most of the time, and no appetite.

Food distressed her after eating. Never drinks any water; but drinks a little tea between meals. Has had no inclination to drink water for a number of years. Passes a very little high-colored urine twice a day. Her complexion is pale; does not sleep at nights. Pulse 108, weak and thready. Temperature 100°. Patient was at once given large quantities of fluids; milk and hot water, palatably seasoned, were prescribed for her, together with such medicines as would aid her in sleeping, and move the bowels Patient soon convalesced, and finally recovered her usual health.

Loss of the sense of thirst, and the abstinence from the necessary amount of water that the system required, was sufficient cause of her embarrassed health. W. F. L.

*Treatment of the Paralytic Affections of Diphtheria.* CORMACK. (*Edinburgh Med. Jour.*, July, 1876.)

In every eleven cases of diphtheria, one is characterized by paralysis. The paralysis is usually first manifested in the inferior extremities; the superior ones, however, soon become involved. The peculiar characteristic is paralysis of the veil of the palate, which occurs as a sequel to diphtheria, but to no other disease. Very frequently the veil of the palate is alone affected, other portions of the bodv escaping. Atrophy in convalescent diphtheritic patients may be due to a protracted disuse of muscles, which, very frequently results in paralysis; where this is true, if prompt and early treatment is instituted, permanent paralysis may be prevented. In convalescence from diphtheria, the dominant condition is asthenia, and to this and anæmia are the paralytic affections attributable, hence the treatment indicated is such as tonics, combined with such local treatment as will improve the nutrition of the muscles involved in the paralysis.

W. F. L.

*Metastases of a simple Bronchocele.* COHNHEIM. (*Centralbl. f. Chir.*, 1877, p. 119.)

At the autopsy of a woman, aged 35 years, who died of decubitus and diphtheria of the colon, a great number of small tumors were found in the lungs and bronchial glands, in the second, third, and fonrth lumbar vertebræ and in the right femur. The structure of all these growths was alike and presented the typic picture of the texture of the thyroid gland. This gland was somewhat enlarged, and its left lobe contained two lumps of a soft gelatinous consistence, one of which sent a small nodule into the lumen of a larger branch of the thyroid vein.

